# Effects of Guajol^®^ ointment synthesized from medicinal smoke condensate of jennet feces on burn wound healing on Wistar rat 

**Published:** 2017-09-15

**Authors:** Farhad Safarpoor Dehkordi, Farhang Tirgir, Yousef Valizadeh

**Affiliations:** Young Researchers and Elites Club, Shahrekord Branch, Islamic Azad University, Shahrekord, Iran

**Keywords:** Burn wound healing, Guajol^ ® ^ointment, Jennet feces, Rat

## Abstract

Application of smoke condensate derived from an indirect heating of jennet feces (Sargin) had been recommended by Iranian ancient scientists as a therapeutic agent. The present study was done to evaluate the healing effects of Guajol^® ^ointment on burn wound in rat. The Guajol^® ^ointment was prepared from the smoke condensate of Sargin samples. Wistar Rats (n = 50) were randomized into six groups including normal saline, silver sulfadiazine and 1.25%, 2.50%, 5.00% and 10.00% concentrations of Guajol^® ^ointment. Under general anesthesia, dorsum of the rats were shaved and burn wounds were created using hot plate. Area of wounds and percent of healing were measured. Normal saline had the highest area of wound, followed by 1.25% Guajol^® ^and silver-sulfadiazine groups. The group treated with 5.00% Guajol^® ^showed the highest percent of healing. Percent of healing in NS, SSD and 5.00% Guajol^® ^ointment groups on day 21 were 38.47%, 75.00% and 98.51%, respectively. Microscopic examination of wounds sections of rats treated with 5.00% Guajol^® ^showed more collagen fibers and fibroblasts cells on day 7. Wounds of 5.00% Guajol^® ^treated group was covered with healthy epithelial and epidermis tissues and hair follicles on day 21. This was the first report of using Sargin to heal the burn wound of rat. Further studies are recommended for investigation of the other effects of Guajol^® ^ointment and its possible application in medicine.

## Introduction

One of the most critical economic issues facing human is burn wound healing. Burn wounds are common injuries all around the world. However, in developing countries burns constitute a major health problem because of the high incidence of severe complications and limitation of financial resources. Incidence rate of burn injuries is about 1.25 million cases per year in developed countries like the United States.^1^ On the other hand, about two million cases are referred to the health care center and hospitals due to the burn injuries annually.^2^ The main aim of burn management and therapy is wound healing and epithelization as soon as possible to prevent infections and to reduce functional and aesthetic effects,^3^ however, the possibility of occurrence of deformities, mis-formation and infection in the site of burn wound are the main concerns facing their application. However, several reports showed the high efficacy of some biochemical agents.^4^

Silver sulfadiazine (SSD) is the topical agent of choice in severe burns and is used almost universally today in preference to compounds such as silver nitrate and mafenide acetate. The SSD cream, while being effective, causes some systemic complications including neutropenia, erythema multiforme, crystalluria and methemoglobinemia.^5^

About 80% of the population of developing countries keep using traditional remedies in their health care.^6^ There are so many medical plants with favorite wound healing effects but these plants are usually found in specific regions and mainly are not available to all people. Smoke produced from natural substances such as plant, animal dung, crop residues and coal, typically burned in open fires have been extensively used in many cultures. Famous ancient physicians have described and recommended use of these materials as substantial medical agents.^7^

Iranian traditional medicine has recommended the use of smoke from complete burning of jennet feces (donkey’s feces, also known as Sargin or *Anbar nesara*) as a strong antimicrobial, antiseptic and flavoring, anti-tumor, anti-inflammatory, anti-nociceptive, anti-bronchitis, wound healing and anti-sinusitis component.^8^^,^^9^ Therapeutic uses of the smoke extracted from the indirect heating of Sargin had been recommended by Avicenna and Rhazes.^8^^,^^9^ Sargin is cheap, affordable and available everywhere. There were so many chemically differences between donkey and human feces. Donkey feces are clean and mainly full from forage and crop residues with antibacterial, anti-oxidative and antifungal effects,^8^^,^^9^ while human feces are full in pathogenic bacteria, fungi and viruses which are dangerous for human health. 

Despite the high values of Sargin, there were no published data on its effects on burn wound healing. Thus, the present study was carried out to determine the effects of Guajol^® ^ointment synthesis from medicinal smoke condensate of Sargin on the healing of burn wounds in rat.

**Ethical consideration. **The experimental protocol of the present study was approved by Ethical and Research Committee of Young Researchers and Elites Club, Islamic Azad University, Shahrekord Branch, Shahrekord, Iran (No. ETH 1201394).

**Preparation of ointment.** From March to May 2015, a total number of 40 Sargin samples were collected from Shahrekord, Chaharmahal va Bakhtiary province, Iran. All Sargin samples were immediately transferred to the Nanoscale Chemical Research Center of the Islamic Azad University of Shahrekord Branch, Iran. All Sargin samples were subjected to direct sunlight until well dried. Then samples were powdered with mechanical pressure induced and were subjected to smoke extraction using indirect heating. Produced smokes were conducted and cooled. Finally, the condensate was reserved for further examinations. Condensates were then subjected to eucerin ointment base and the ointment was prepared. Based on the idea of inventors of this ointment, Guajol^® ^(registered name for a product) was chosen for its name. This ointment was registered in the Food and Drug Administration (FDA) of The Islamic Republic of Iran with production license number of S-94-0436.

**Gas chromatography/mass spectrometry. **Volatile components of smoke condensate of Sargin were identified using gas chromatography–mass spectrometry (GC-MS). The chemical composition of Sargin smoke was analyzed using gas chromatograph (Agilent 7890 GC; Agilent Technologies, Palo Alto, USA) with a HP-5MS 5% phenylmethylsiloxane capillary column (30.00 m × 0.25 mm, 0.25 µm film thickness). Oven temperature was kept at 60 ˚C for 4 min initially, and then raised at the rate of 4 ˚C per min to 260 ˚C. Injector and detector temperatures were set at 290 ˚C and 300 ˚C, respectively. Helium was used as the carrier gas at a flow rate of 2 mL min^-1^. Totally, 0.10 µL of samples were injected manually in the split mode. Peaks of area percent were used for obtaining quantitative data. The GC was coupled to an Agilent 5975 C (Agilent Technologies) mass selective detector. The EI-MS operating parameters were as follow: Ionization voltage of 70 eV and the ion source temperature of 200 ˚C. Retention indices were calculated for all components using a homologous series of n-alkanes (C5 - C24) injected in conditions equal to samples. Identification of smoke components was accomplished based on comparison of their retention times with those of authentic standards and their mass spectral fragmentation patterns (willey/ ChemStation data system) and the calculated retention indices with the retention indices compilation of the NIST Mass Spectrometry Data Center.^10^

Animal models and experiment. All animals were obtained from Pasteur Institute in Iran. Male Wistar rats (n = 50) weighing 150 to 200 g with average age of 10 weeks old were purchased. Animals were kept under standard conditions at room temperature (22 ˚C and 50% humidity) with 12/12 hr light/dark cycle. Animals had laboratory food and water, *ad libitum*. The rats were anesthetized by intraperitoneal injection of 50 mg kg^-1^sodium thiopental (Sigma-Aldrich, Dorset, UK). The dorsum was shaved using an electrical clipper and 70% alcohol was used to disinfect the area. General anesthetized rats were kept in prone position. A deep second degree burn wound was induced by a hot plate (a standard area of 2.50 × 2.50 cm) heated for 5 min in boiling water (temperature of boiling was measured according to its relation with the altitude) and placed for 10 sec on the skin of rats. The pressure exerted on the animal skin corresponded to the mass of 51 g of aluminum bar used in the burn induction.

**Study the healing effects of ointment.** In order to study the wound healing effects of the Guajol^® ^ointment, four different concentrations were made using the absolute ethanol (Merck, Darmstadt, Germany) and eucerin ointment base (Shahtalebi Co., Isfahan, Iran) (1.25%, 2.50%, 5.00% and 10.00%). Group 1 wounds (control group) were only treated with normal saline (NS) in each dressing round. Group 2 was treated with Silver sulfadiazine 1.00% (SSD, Sina-Daru Inc., Tehran, Iran). Groups 3, 4, 5 and 6 were treated with 1.25%, 2.50%, 5.00% and 10.00% concentrations of the Guajol^® ^ointment, respectively. Wound area of each rat was occluded with a 2.50 × 2.50 cm piece of sterile gauze that was moistened with sterile PBS. This layer was further occluded with a 5 × 5 cm piece of plastic sheet.

All wounds were washed with normal saline in on daily basis, and wounds in treatment groups received their appropriate cream and were dressed. In order to quantify the rate of wound healing, the size of lesions was determined on 1, 10 and 20 days after burn injury.^11^ The wound area was recorded as cm^2 ^(using the AutoCad software). Percent of wound surface area and also percent of healing were measured formulas follow:

*Percent of wound* *surface* *area= (* *Area of wound in certain day/ Area of wound in first day) × 100*

and

*Percent of healing = 100 – percent of wound surface area*

**Histological examinations.** All histological procedures were performed by an independent blind observer. From each sample in each day, two slides were prepared using sterile scalpel and tissue forceps. Burned skin tissue samples were taken for histological studies with a small excision containing part of the wound area. Tissue samples were fixed in 10% formalin solution. Paraffin embedded tissue section of 4 μm were prepared and stained with hematoxylin and eosin (H & E). Light microscopy was used to assess pathological changes.^12^

**Statistical analysis.** Data were transferred to the Excel software and reported as mean and standard deviation (Mean ± SD). Statistical comparisons between groups were carried out using SPSS (version 19.0; SPSS Inc., Chicago, USA). One-way ANOVA test was used to analyze data. A *p* value ≤ 0.05 was considered as statistically significant.

## Results

Table 1 shows the constituents of the medicinal smoke condensate of Sargin. Totally, nine different components were detected in the smoke condensate of the Sargin. Frequency of detected components were 69.86%. P-Xylene (24.88%), Syringol (11.90%), m-Xylene (6.54%), o-Xylene (5.19%) and o-Methoxyphenol (Guaiacol; 6.02%) were the most commonly detected chemical components in the smoke condensate of Sargin. 

**Fig. 1:**
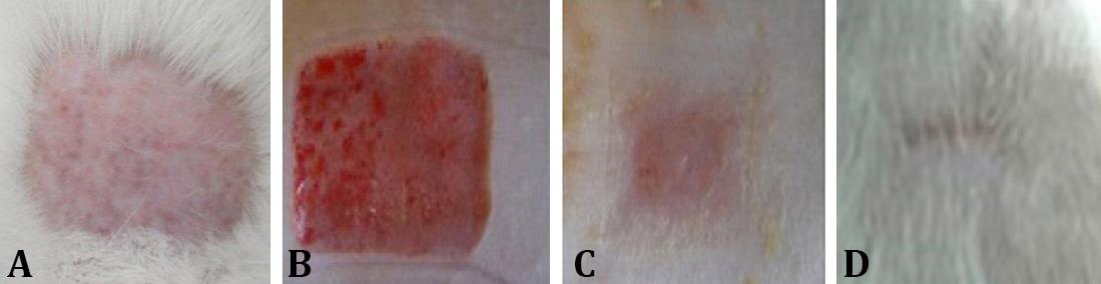
Macroscopic examination of the appearance of second-degree burns in Wistar rats. A) Animals skin after shaving; B) Thermal burn lesion with mild edema (day 0); C) Injured tissue on day 7 after burn induction, presence of granulation tissue in the center of the lesion with a second discreet crust and formation of scar tissue at the edge; D) Injured tissue on day 21 after the burn induction with incomplete healing

Figure 1 shows the macroscopic appearance of burn wounds through 21 days of treatment of rats with 5.00% Guajol^® ^ointment. The processes of granulation, formation of scar and incomplete healing are also shown. 

**Fig. 2:**
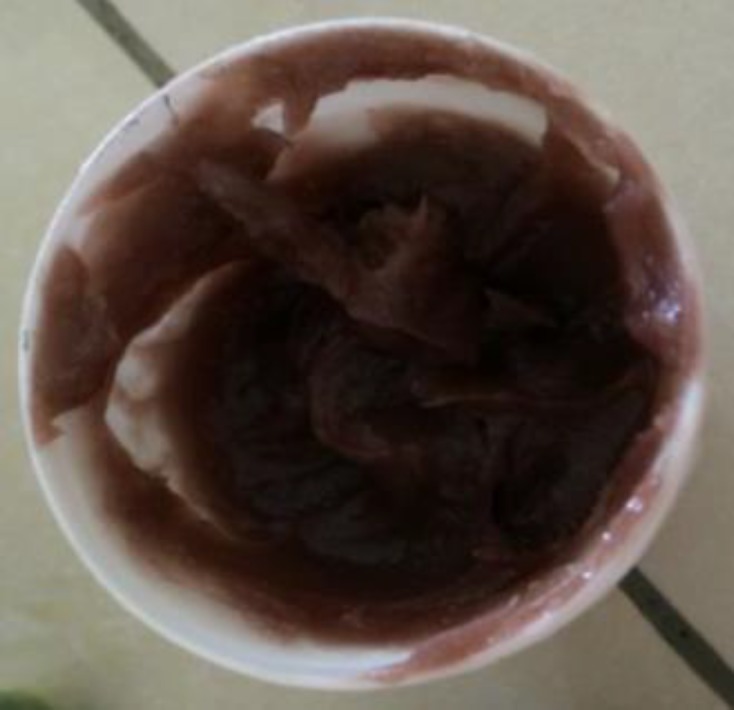
Appearance of the produced ointment as the Guajol^®^.

Figure 2 represents the appearance of the Guajol^®^ ointment. Table 2 represents the percent of wound and also percent of healing in each studied groups. We found that the rat treated with NS had the highest area of wound on day 7 (10.70 ± 1.51 cm^2^) and day 21 (5.40 ± 0.84 cm^2^). Area of the wounds which were treated with 1.25% Guajol^® ^were high on day 7 (9.50 ± 1.30 cm^2^) and day 21 (4.60 ± 0.61 cm^2^). Area of the wounds which were treated with SSD were also high on day 7 (8.30 ± 1.24 cm^2^) and day 21 (3.20 ± 0.53 cm^2^). Percent of wound in the rats treated with NS, SSD and 5.00% Guajol^® ^ointment in day 21 were 41.53%, 25.00% and 1.49%, respectively. Percent of healing in the group of rats treated with 5.00% Guajol^® ^on days 0, 7 and 21 were 0.00%, 71.65% and 98.51%, respectively which were significantly higher than those of other treatment groups (*p *≤ 0.01). The group treated with NS had the lowest percent of healing among all six groups (*p *≤ 0.05). Rats treated with 5% Guajol^®^ ointment had the highest levels of wound healing and lowest levels of wound area (*p *≤ 0.05).

**Table 1:** Chemical components of the medicinal smoke condensate of jennet feces

**Compound**	**RT**	**KI**	**(%)**
**o-Xylene ( alpha-Methyltoluene )**	3.99	931	5.19
**p-Xylene**	4.11	937	24.88
**m-Xylene**	4.52	958	6.54
**dl-Limonene (Mentha-3,8-diene (para)**	7.61	1086	4.14
**Mentha-2,8-dien-1-ol (cis-para)**	8.97	1132	1.50
**o-Methoxyphenol (Guaiacol)**	9.38	1145	6.02
**2,3-Dimethylphenol (2,3-Xylenol)**	11.83	1224	1.51
**Syringol (2,6- Dimethoxyphenol)**	28.58	1787	11.96
**Hexadecanol**	30.62	1877	1.76
**Total**	63.52		

**RT = **Retention time;** KI = **Kováts index.

**Table 2:** Total area of wound, percent of wound and percent of healing in each studied group in various days

**Groups**	**Days of test**	**Area of wound (cm** ^2^ **)**	**Percent of wound **	**Percent of healing**
**Group 1 (Normal Saline)**	0	13.00 ± 2.42^a^*	100^a^	0.00
	7	10.70 ± 1.51^a^	82.30^a^	17.70^a^
	21	5.40 ± 0.84^b^	41.53^b^	58.47^b^
**Group 2 (Silver sulfadiazine)**	0	12.80 ± 2.68^a^	100^a^	0.00
	7	8.30 ± 1.24^b^	64.84^a^	35.16^b^
	21	3.20 ± 0.53^c^	25.00^c^	75.00^d^
**Group 3 (1.25% Guajol** ^®^ **)**	0	13.70 ± 2.68^a^	100^a^	0.00
	7	9.50 ± 1.30^a^	69.34^a^	30.66^b^
	21	4.60 ± 0.61^c^	33.57^b^	66.43^d^
**Group 4 (2.50% Guajol** ^®^ **)**	0	13.40 ± 2.93^a^	100^a^	0.00
	7	7.10 ± 1.17^b^	52.98^a^	47.02^b^
	21	1.70 ± 0.48^d^	12.68^c^	87.32^e^
**Group 5 (5.00% Guajol** ^®^ **)**	0	13.40 ± 1.42^a^	100^a^	0.00
	7	3.80 ± 0.76^c^	28.35^b^	71.65^c^
	21	0.20 ± 0.10^e^	1.49^d^	98.51^d^
**Group 6 (10.00% Guajol** ^®^ **)**	0	12.80 ± 2.13^a^	100^a^	0.00
	7	7.30 ± 1.25^b^	57.03^a^	42.97^b^
	21	2.00 ± 0.56^c^	15.62^c^	84.38^e^

**abcde:** Different superscript letters in each column represent significant differences at *p *≤ 0.05.

**Fig. 3:**
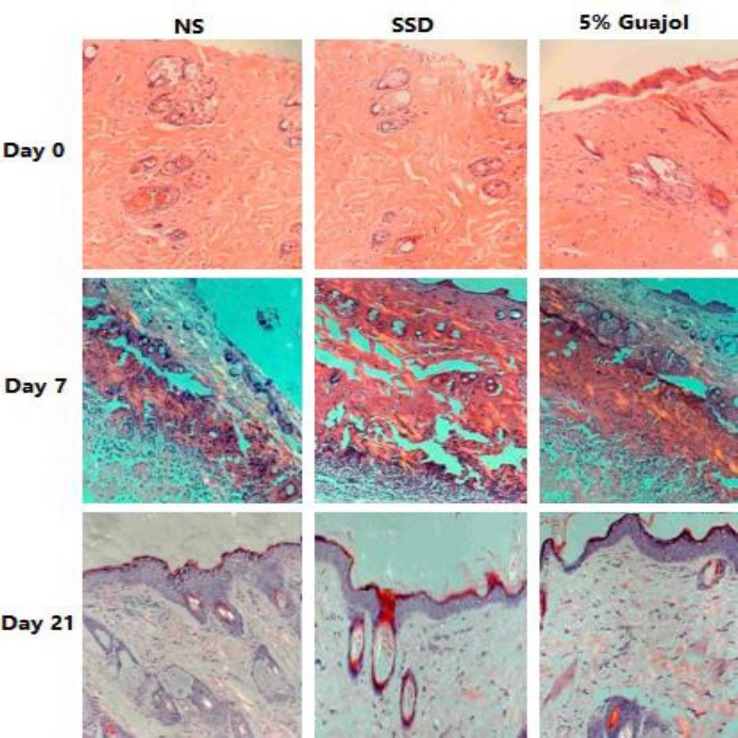
Results of the microscopic examination of burn wound healing using a hematoxylin–eosin staining in rat model. On the first day after burn, the pathological sections showed that there were neither dermis nor epidermis in all studied groups with higher levels in the group treated with 5.00% Guajol^®^. At the day 7, the scabs were dropped, and some new epithelial tissues were observed with higher levels of epithelization on the group treated with 5.00% Guajol^®^ and finally on the day 21, the wound was covered with healthy epithelial tissue and the new epidermis, and generated the sebaceous and sweat gland with complete tissue re-epithelialization, fibroblastic proliferation, presence of modeled dense collagen mesh, and moderate fibrosis. The levels of healing in the 5.00% Guajol^® ^group was entirely higher than SSD and NS groups (100×).

Figure 3 represents the microscopic features in the H & E staining of burn wounds in each studied groups. Histological sections taken from the rats treated with 5.00% Guajol^® ^ointment showed more collagen fibers and fibroblasts cells on day 7 of evaluation compared to the control group. After 21 day, the wound treated with 5.00% Guajol^® ^was almost covered with healthy epithelial tissue and the new epidermis.

These rats also showed complete epithelialization in the burn injury area, and the sebaceous glands in the section were clear. Complete tissue re-epithelialization, fibroblastic proliferation, presence of modeled dense collagen mesh, and moderate fibrosis were another important finding in the sections taken from the rats treated with 5.00% Guajol^® ^ointment. All of these properties were incomplete in other studied groups. 

## Discussion

Burn is the most prevalent and important form of trauma. Burn is a causative agents of a lot of morbidities and mortalities in the world.^13^ Burn therapeutic options can accelerate skin healing and help prevent from the occurrence of infection.^13^ Studies have confirmed that infections in the sites of injured skin are major reasons of mortality in patients with widespread burns. Therefore, application of topical antimicrobial agents can reduce the risk of wound infections and shorten the period of treatment in patients with burn wounds.^13^ To the best knowledge of the authors, the present investigation was the first report of application of smoke condensate of Sargin as a good burn wound healing agent in rat all around the world. The animals treated with 5.00% Guajol^® ^had the highest healing effects compare to other groups. The area of wound in the rats treated with 5.00% Guajol^® ^on day 7 was nearly equal with that of SSD group on day 21 which was considerable. We found that the percent of healing in the rats treated with NS, SSD and 5.00% Guajol^® ^at the end of day 21 were 38.47%, 75.00% and 98.51%, respectively. Healing effects of the 2.50% and 10.00% concentrations of the Guajol^® ^ointment were also higher than SSD. However, the main reason for the high healing effects of the Guajol^® ^was unknown but it seems that aromatic hydrocarbons and xylene-based components which were produced due to the indirect heating of Sargin were the main reason. The results of the macroscopic examination were also approved by the microscopic examinations. Totally, there were no inflammatory reactions, presence of neutrophils and bleeding in the group treated with 5.00% Guajol^® ^after 21 days of experiment. In addition, differentiated tissues in the epidermis, dermis, hypodermis and the newly formed hair follicles were significant in the histological sections of rats treated with 5.00% Guajol^® ^which was entirely higher than those of SSD.

Parvin *et al*. showed that some nosocomial infectious agents such as *Staphylococcus aureus* and *Pseudomonas aeroginosa* were sensitive to the smoke extracted from an indirect heating of jennet feces.^14^ *Staphylococcus aureus* is one of the most important causes of burn and wound infections al-around the world.^15^^-^^17^ The main reasons for the higher healing effects of this invented ointment could be related to the high antimicrobial effects of Guajol^® ^on two important causes of infection in the sites of burn wound (i.e. *S. aureus* and *P.* *aeroginosa*)^14^^,^^18^ and also occurrence of high antibiotic resistance of these pathogenic agents against SSD.^5^^,^^19^ High burn wound healing effects of cow^20^ and goat^21^ dungs have been reported previously. Application of smoke, hot metal burns, dung and animal and plant products as a good therapeutic option for healing of wounds and burns is common among some African countries.^22^ Shaikh and Shaikh, showed that the ash taken from the burning of buffalo dung had a high wound healing effects on rabbit.^23^ They showed that consistent healing was observed in all the experimental wound sites, which was comparatively more rapid than the control wound site. The healing was complete on eleventh day only with charcoal ash whereas for dung-cake ash and wood ash, the completion time was approximately 13 days. Justin-Temu *et al*. reported that application of agents such as cow and goat dungs may contaminate the wounds and lead to development of serious infections including tetanus and septic wounds thus prolonging the stay in hospital for treatment.^24^ In despite of other types of dung, Sargin has a high antimicrobial effects which can clean the site of burn wound and inhibit from occurrence of infection.^14^

The present study suggested that four different concentrations of Guajol^® ^ointment were able to improve the healing process of burn wound. Although it may be argued that the higher effects of 5.00% Guajol^® ^than 2.50% and 1.25% concentrations could be due to the higher concentration of ethanol, as a germicide agent, for its production. In fact, 10.00% medication has less volume/ weight of solvent/base in comparison with 5.00% medication. The results of 10.00% Guajol® group which harbored the higher levels of ethanol was lower than 5.00% Guajol^® ^group. The results obtained from the group treated with 10.00% Guajol^® ^showed that ethanol could not accelerate the wound healing. Another reason may be due to the higher amount of effective chemical components in 5.00% ointment compared to 1.25% and 2.50% groups. Lower healing effects of 10.00% Guajol^® ^ointment compared to 5.00% one might be due to the higher toxic level of 10.00% Guajol^® ^ointment. It seems that 5.00% Guajol^®^ ointment at was in the therapeutic margin, however, at 10.00% concentration reached to the toxic level.

The final important point was the sense of usage of Guajol^® ^ointment. As we said, this ointment is taken from the indirect heating of the jennet feces. Therefore, there were no direct contact with the jennet feces. In addition, application of high temperature in the processing of primary material of the ointment made it safe and clean from any pathogenic and dangerous agents. Chemical analyses of components in the ointment did not show any dangerous, odorous and even disgusting materials. It is strong in anti-oxidative, anti-bacterial and anti-fungal agents which cannot be compared with human feces. In addition, there are so many kinds of ointment, tablets and other type of therapeutic options which their main sources and effective materials are unknown and may even be more disgusting. In keeping with the effects of the Guajol^® ^ointment and also its safe and clean processing, there are no concerns for considering the use of this ointment as a good burn wound healing agent. 

In conclusions, we found that the medicinal smoke condensate of Sargin at a 5.00% concentration could accelerate the healing of second-degree burn wound in rats. Complete healing of burn wounds on day 21 with respect to the low levels of inflammatory reactions, neutrophils and bleeding and high presence of differentiated tissues and hair follicles in the skin layers were confirmed the regenerative effects of 5.00% Guajol^®^ ointment. Further studies are recommended for evaluation of the other effects of Guajol^® ^ointment and also the possibility of its application in the medicine. 
